# CloVR-Comparative: automated, cloud-enabled comparative microbial genome sequence analysis pipeline

**DOI:** 10.1186/s12864-017-3717-3

**Published:** 2017-04-27

**Authors:** Sonia Agrawal, Cesar Arze, Ricky S. Adkins, Jonathan Crabtree, David Riley, Mahesh Vangala, Kevin Galens, Claire M. Fraser, Hervé Tettelin, Owen White, Samuel V. Angiuoli, Anup Mahurkar, W. Florian Fricke

**Affiliations:** 1 Institute for Genome Sciences, Baltimore, MD USA; 2 Department of Microbiology and Immunology, University of Maryland School of Medicine, Baltimore, MD USA; 3 Department of Epidemiology, University of Maryland School of Medicine, Baltimore, MD USA; 4 Department of Nutrigenomics, University of Hohenheim, Stuttgart, Germany

**Keywords:** Comparative genomics, Microbial genomics, Automated analysis, Whole-genome alignment, Bioinformatics resource, Virtual machine, Cloud computing

## Abstract

**Background:**

The benefit of increasing genomic sequence data to the scientific community depends on easy-to-use, scalable bioinformatics support. CloVR-Comparative combines commonly used bioinformatics tools into an intuitive, automated, and cloud-enabled analysis pipeline for comparative microbial genomics.

**Results:**

CloVR-Comparative runs on annotated complete or draft genome sequences that are uploaded by the user or selected via a taxonomic tree-based user interface and downloaded from NCBI. CloVR-Comparative runs reference-free multiple whole-genome alignments to determine unique, shared and core coding sequences (CDSs) and single nucleotide polymorphisms (SNPs). Output includes short summary reports and detailed text-based results files, graphical visualizations (phylogenetic trees, circular figures), and a database file linked to the Sybil comparative genome browser. Data up- and download, pipeline configuration and monitoring, and access to Sybil are managed through CloVR-Comparative web interface. CloVR-Comparative and Sybil are distributed as part of the CloVR virtual appliance, which runs on local computers or the Amazon EC2 cloud. Representative datasets (e.g. 40 draft and complete *Escherichia coli* genomes) are processed in <36 h on a local desktop or at a cost of <$20 on EC2.

**Conclusions:**

CloVR-Comparative allows anybody with Internet access to run comparative genomics projects, while eliminating the need for on-site computational resources and expertise.

**Electronic supplementary material:**

The online version of this article (doi:10.1186/s12864-017-3717-3) contains supplementary material, which is available to authorized users.

## Background

With the latest advancements in high-throughput sequencing, a deluge of annotated bacterial genomes has been submitted to public repositories, such as the National Center for Biotechnology Information (NCBI) Reference Sequence Database (RefSeq, https://www.ncbi.nlm.nih.gov/refseq/). Thousands of new genomes continue to be sequenced as part of large-scale bacterial genome projects, such as the Genomic Centers of Infectious Diseases (https://www.niaid.nih.gov/research/genomic-centers-infectious-diseases). Automated bioinformatics support for these and other projects, including assembly, gene finding and functional annotation, are provided by computerized services, such as RAST (http://rast.nmpdr.org/), IMG/M (https://img.jgi.doe.gov/) or CloVR (http://clov.org). However, less support is available for downstream comparative analyses of the previously and newly generated annotated microbial genome sequence data. Consequently it can be difficult for researchers who are unfamiliar with bioinformatics sequence analysis, to determine the best and most suitable analysis protocol to address their research question, to select and apply the corresponding bioinformatics tools, and to identify IT resources to access, store and process large amounts of associated sequence data. Therefore, access to bioinformatics tools and infrastructure for comparative analysis has become a bottleneck in the widespread use of available genome sequence data.

We describe CloVR-Comparative, an open-source, automated, easy-to-use bioinformatics pipeline for comparative genome sequence analysis. CloVR-Comparative facilitates the integration of user-provided and publicly available datasets into the analysis workflow and does not depend on local access to high-performance computing resources, as it provides optional support for using online cloud computing resources. Thus, CloVR-Comparative allows researchers to run analyses independent of local bioinformatics expertise and resources, thereby increasing the utility of public genome databases to the broader research community and facilitating follow-up research on available sequence data.

## Implementation

### Cloud Virtual Resource (CloVR) integration

The comparative genome analysis protocol described here was installed on the Cloud Virtual Resource (CloVR) (http://clovr.org), a bioinformatics framework for microbial sequence analysis [1, 2]. CloVR is an open-source virtual appliance (http://sourceforge.net/projects/clovr/) that includes automated bioinformatics workflows for microbial genomics applications with optional support for online cloud computing services. The CloVR virtual appliance can be installed locally on computers running Windows, OS X or Linux/Unix operating systems using freely available virtual machine players such as VMware (http://www.vmware.com/) or VirtualBox (https://www.virtualbox.org/). CloVR provides full functionality over all included microbial sequence analysis pipelines, including the CloVR-Comparative pipeline (starting with CloVR version “clovr-standard-2014-10-07-21-11-54_vdi.tgz”), without requiring additional installations or configurations. In addition, it also supports installation on online cloud computing service, including EC2, the Amazon Elastic Compute Cloud (https://aws.amazon.com/ec2).

CloVR includes a graphical user interface (GUI), called CloVR Dashboard, which is accessible via a web browser and provides users with an overview of input and output data that is available to and from CloVR. In addition, the CloVR dashboard allows users to upload and download data, select and configure individual analysis pipelines and monitor pipeline progress.

### CloVR-Comparative input

**Fig. 1:**
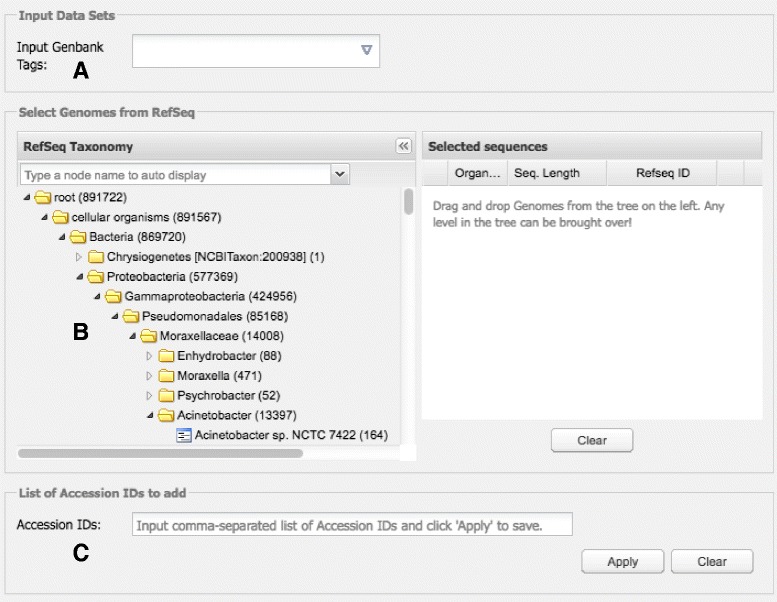
CloVR-Comparative configuration screen. Three options are available to the user to identify and select annotated genome sequence data as input for CloVR-Comparative: **a** Using uploaded GenBank files or GenBank files generated by the CloVR-Microbe protocol both of which can be specified by so-called “tags” as described in the CloVR documentation [1]; **b** Through drag-and-drop in the searchable interactive interface that lists genomes available from RefSeq in a taxonomic tree format; and **c** By specifying a list of comma-separated GenBank accession numbers

CloVR-Comparative requires annotated genomes as input in GenBank format, as generated from the CloVR-Microbe pipeline [3] or by annotation services, such as the Institute for Genome Sciences’ Analysis Engine (http://www.igs.umaryland.edu/research/bioinformatics/analysis/), or as available from the NCBI RefSeq archive (https://www.ncbi.nlm.nih.gov/refseq/). Genome sequence data can be directly uploaded to CloVR or added as a list of GenBank accession numbers via the CloVR-Comparative configuration window (Fig. 1). This configuration window also includes an interactive user interface with a tree-based presentation of genomes available from RefSeq using the NCBI taxonomy (http://www.ncbi.nlm.nih.gov/taxonomy). Taxonomy (in *.obo format) and RefSeq genomes (in GenBank format) are downloaded from NCBI and combined into a compressed binary file, which is used by the JavaScript application framework ExtJS (https://www.sencha.com/products/extjs/) to display an interactive and searchable taxonomic tree of all bacterial species. Taxonomy and genomes are updated frequently. The interface allows users to search, select and include RefSeq genomes into the CloVR-Comparative analysis with drag-and-drop mouse gestures.

The input GenBank files should contain gene calls, annotations and genomic sequence, as CloVR-Comparative analysis results depend on the accuracy and quality of the annotation for the prediction of clusters of orthologous genes and the downstream identification of core, shared and unique CDSs and SNPs. Both draft genomes, i.e. input data consisting of multiple annotated sequences with the same organism name, as well as complete genomes are valid input for the pipeline.

### CloVR-Comparative protocol overview

**Fig. 2:**
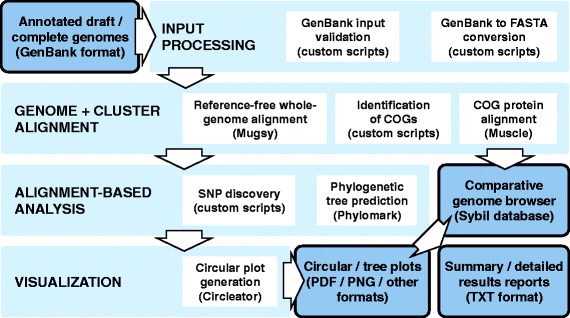
Overview and flowchart of CloVR-Comparative. Input data in the form of annotated genomes in GenBank format is first validated and converted into other file formats, then used in whole-genome alignment (WGA) with Mugsy and alignment of translated CDS with MUSCLE to determine COGs. WGAs are used to identify SNPs and to predict phylogenetic relationships based on core genomic regions with Phylomark. From the results individual circular plots are generated for each input genome. The analysis output is loaded into a Sybil database to provide searches of comparative genome data in a web browser, summary and detailed results reports, and tree and circular figures

The CloVR-Comparative pipeline consists of four major modules: Input Processing, Genome and Cluster Alignment, Alignment-based Analysis, and Visualization (Fig. 2). Several components within each of the four modules are executed in parallel to improve performance and efficiency in multi-processor environments. The modules are described in detail below. A list of custom scripts that are part of CloVR-Comparative is provided as part of Additional file 1: Table S1.

### Input processing

The first input processing step is the validation, formatting and preparation of the input files for whole-genome alignment. The most important step within this module is the GenBank check that validates the input files and automatically corrects features that do not conform with the GenBank format or removes features that are not relevant for the analyses performed in CloVR-Comparative. Examples of corrected features are malformed locus identifiers and accession numbers longer than 16 characters in length. Removed features include pseudogenes. Sequences are extracted from multiple GenBank files belonging to the same source organism and used to create a single multi-FASTA genome sequence file. A mapping file, i.e. a tab-delimited text file, with all gene calls and annotations from all the input genomes is generated for the prediction of clusters of orthologous genes.

### Multiple whole-genome alignment

Whole-genome alignments are performed on multiple genome FASTA files with Mugsy [4]. Mugsy is a rapid, reference-free multiple whole-genome DNA alignment tool, which employs a whole-genome pairwise aligner, NUCmer [5], to identify homology, rearrangements and duplications between closely related genomes. Mugsy output is a multiple alignment file in MAF format.

The Mugsy alignment in MAF format along with the annotation-containing mapping file created during input processing is passed to the Mugsy-Annotator program [6]. Mugsy-Annotator uses the alignment to identify CDSs that are conserved in both nucleotide sequence and genome location. Core, shared, and unique CDSs are identified using Mugsy-Annotator. *Core CDSs* are defined as present in all input genomes and aligned to each other in the whole-genome alignment. *Shared CDSs* are aligned in at least two but not all input genomes. *Unique CDSs* are part of whole-genome alignments and thus should only be present in a single input genome. However, the results of the pipeline completely rely on the annotations provided in the input GenBank files. Since Mugsy-Annotator only aligns genes that are conserved in genome position, follow up analysis, including additional genome searches, should be performed on unique genes to determine their presence or absence in the other genomes. The Mugsy-Annotator output consists of two files, a formatted clusters of orthologous genes (COGs) file (mugsyoutput.cog), listing the predicted clusters of orthologous genes between the genomes, and a raw COGs file (mugsyoutput.raw) with more details for each cluster, including all CDS and annotations from each genome within the cluster.

### Alignment-based comparative analysis

The COGs predicted by Mugsy-Annotator are translated into protein sequences and aligned using MUSCLE [7] with default parameters. The resulting multiple protein alignments are provided for each COG in GCG Multiple Sequence File (MSF) alignment format and are also available to view in the Sybil comparative genomics viewer [8], which is included in CloVR as a visualization and browsing tool for the results of CloVR-Comparative and described later.

SNPs from the whole-genome alignment are detected with a custom Perl script (mugsy_callsnps.pl; https://github.com/jorvis/ergatis/blob/master/src/perl/mugsy_callsnps.pl). It processes the MAF multiple whole-genome alignment file to report SNP positions within each genome, i.e. locations where single nucleotides differ between at least two aligned input genomes. Subsequently, another custom Perl script (summarize_mugsy_comparative_pipeline.pl; https://github.com/jorvis/ergatis/blob/master/src/perl/mugsy_callsnps.pl) is used to annotate SNP positions in each of the genomes using information from the raw COGs file and the annotation mapping file. A tab-delimited, annotated SNP file as well as a variant call format (VCF) file are generated for each of the input genomes listing all SNP positions, including accession of the genome/contig, SNP position and SNP nucleotide variants. For these individual reference-based SNP files a SNP is defined as a nucleotide position where at least one other genomes of the alignment contains a different nucleotide than the input or reference genome.

Phylogenetic relationships between the input genomes, derived from identifying all positions with polymorphisms within the whole-genome alignment, are predicted using components of Phylomark [9], which include steps to select and concatenate core genome fragments, curate the alignment and remove columns with gaps (i.e. indels in any of the input genomes), low diversity and non-contiguous genomic regions, and predict a phylogenetic tree with FastTree2 [10]. The phylogenetic relationship between all input genomes is represented as a tree file in the Newick format (.tree) and as a figure in PDF and SVG format.

### Summary report and visualization of analysis results

**Fig. 3:**
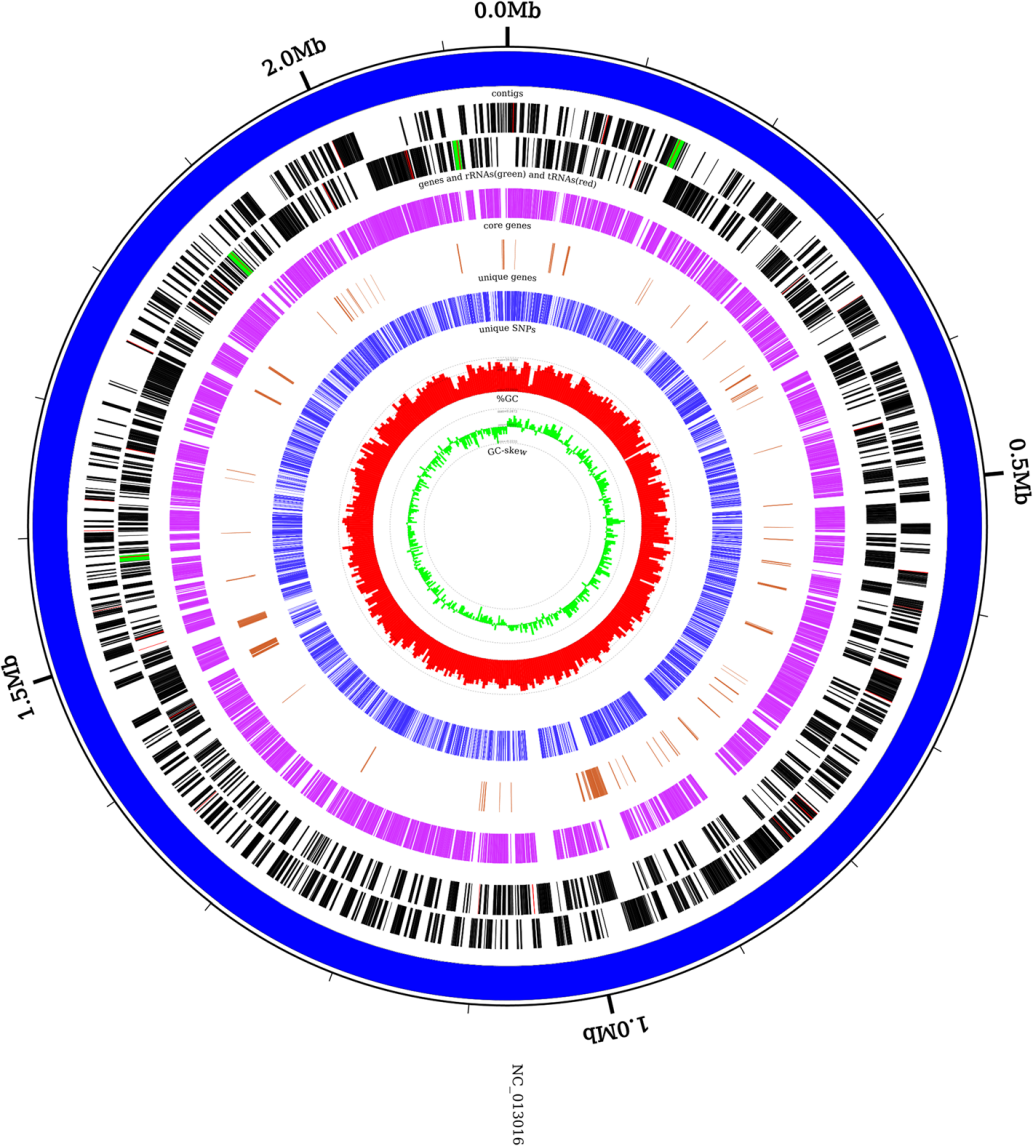
Example of a circular figure output. The figure was generated with Circleator using the example test dataset from the project website as input. It uses *Neisseria meningitidis alpha14* (GenBank accession number: NC_013016) as a reference and depicts from outside to inside (1) complete genome (contigs of draft assemblies would be sorted by size); (2, 3) CDSs on forward and reverse strands; (4) core CDSs, defined as COGs that are shared between all input genomes; (5) unique CDSs that are only present in the reference genome (i.e. *S.* Typhimurium LT2 in this case); (6) unique SNPs, defined as being part of the core genome shared between all input genomes but containing a nucleotide in the reference genome that is different from all other input genomes; (7) G + C content in percent with maximum value shown as *gray dotted line*, calculated using non-overlapping windows of 5kbp length; and (8) GC skew, with maximum value shown as *gray dotted line*, calculated as (G - C) / (G + C) where G and C are nucleotide counts over non-overlapping windows of 5kbp length

The CloVR-Comparative output puts a strong emphasis on the visualization and summary of the analysis results, using the following tools and custom Perl scripts:

1. Sybil: Sybil is an open-source, web-based software for the visualization and analysis of comparative genome data (http://sybil.sourceforge.net/), with a particular focus on protein and gene cluster information [8]. Sybil enables interactive visualization of syntenic regions and synteny breakpoints, genome annotations, conservation and variation among genomes, “missed” gene models etc. The CloVR-Comparative output includes a downloadable Sybil database archive file that can be easily deployed on any CloVR virtual machine running locally or on the cloud, making Sybil-supported comparative analysis portable across CloVR instances and facilitating collaborative projects based on the CloVR-Comparative results. In addition, a Sybil instance is automatically launched on the virtual machine that runs CloVR-Comparative, together with a link on the CloVR Dashboard to the Sybil website that is associated with this instance.
2. Circleator: Circleator is a Perl-based application for generating publication-quality, circular genome figures [11]. The results of the comparative genome analysis are represented by separate, individual circular figures for all input genomes as references, including complete and draft genomes. Circleator output figures provide an overview of the reference genome assembly (complete or draft), all CDSs on both strands, core and unique CDSs and SNPs, G + C content and GC skew. Figure 3 gives an example of the Circleator output with a detailed description of all represented features.
  Information on genome and contig lengths and genome sequence data for the calculation of G + C content and GC skew are extracted from the GenBank input files, whereas information on shared and unique CDSs and unique SNPs is taken from the alignment-based comparative analysis results. Output figures are created in PNG and PDF file formats.
3. Summary report files: Two tab-delimited summary report text files are generated using custom Perl scripts. This includes an overview of the analysis input and results (“summary_report”), which lists all input genomes together with a description of their main features and analysis results, i.e. number of contigs, total and unique genome or cumulative contig length, total, core and unique CDSs. This file also reports references for all tools that are part of the CloVR-Comparative pipeline. An example of a comparative summary report is shown in Additional file 2: Table S2.
  In addition, individual summary report files are generated for each input genome (“summary_file”) that provide an extensive description of the comparative analysis results using this input genome as a reference.

## Results and discussion

### Overview

CloVR-Comparative was built to facilitate access to complex comparative microbial analyses for the inexperienced user while also maintaining high functionality for the experienced user. Towards this goal, the CloVR-Comparative output was organized into the following four groups:

#### Summary reports

To provide the user with a quick overview and fast way to review the success of the analysis, CloVR-Comparative generates a summary report for each pipeline run, which also includes references to the original publications on individual analysis components as background information for interested users. The summary report lists the alignment-based estimated core genome length shared between all compared genomes, and the genome length, total number of CDSs and unique CDSs per input genome. This information allows for the quick identification of potential problems with the analysis run and/or the input genomes. For example, a short core genome length could indicate problems with the whole-genome alignment due to short, fragmented, incomplete or divergent input sequences. Low or high numbers of total or unique CDSs in a particular input genome could indicate problems with the gene prediction in the corresponding input file.

#### Graphical representations

To quickly and intuitively evaluate the comparative output and generate hypotheses for downstream analyses CloVR-Comparative automatically generates a set of graphical representations of selected analysis results. Phylogenetic trees are a common and widely used tool in comparative genomics to depict the evolutionary relationships, i.e. to place a bacterial isolate within the framework of known strains and isolates [12]. Phylogenetic trees can be used to identify the closest genomic relative of a particular bacterial isolate, which can be clinically relevant, as for example in the case of *Escherichia coli* O104:H4 strains associated with the 2011 German foodborne disease outbreak, which showed similarity to enteroaggregative *E. coli* (EAEC) while harboring the Shiga toxin gene cluster typically not found in this genomic context [13–15]. Furthermore, phylogenetic trees can inform downstream comparative analyses, if for example members of an evolutionarily closely related subgroup are identified within a bacterial species [16].

Circular figures represent another widely used graphical output for comparative analyses, which are typically based on choosing one input genome as a reference to highlight additional or missing features from other input genomes. CloVR-Comparative generates separate circular figures for each input genome that show contigs and all CDSs for this genome sequence, as well as core and unique CDSs and SNP positions compared to all other input genomes.

#### Interactive browsing

Comparative genome projects can greatly benefit from interactive, collaborative tools suitable for detailed results browsing, such as the comparative analysis of individual genes or operons in their genomic context across all compared genomes. As part of the CloVR package, CloVR-Comparative is distributed together with an installation of the Sybil comparative genome viewer [8]. CloVR-Comparative automatically generates a Sybil database, which is directly linked from the CloVR Dashboard and opens Sybil as an interactive web browser application. This link can be shared between multiple users, allowing for simultaneous online data browsing. Alternatively, the database file generated by CloVR-Comparative can be shared and opened on any computer running the CloVR virtual appliance, further supporting collaborative projects across separate physical locations.

#### Detailed analysis files

Additional results are generated as detailed text files to be searched for more specific, user-relevant analysis results. These include the whole-genome alignments and a summary file for each input genome, which lists every CDS from that genome together with start and stop positions, annotations, the identified orthologs from other input genomes (if present), and all SNPs found in these CDSs, including the corresponding variants in orthologs from other genomes.

### Analysis examples

**Fig. 4:**
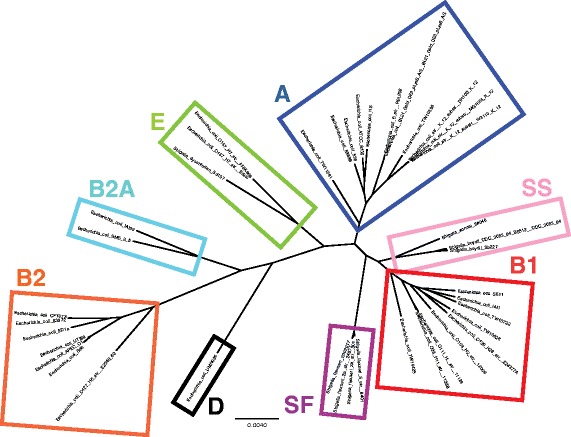
Whole-genome alignment-based phylogenetic tree of 40 *E. coli* genomes from different phylogroups. Reference genomes from eight *E. coli* strains (see [9] for GenBank accession numbers) were used as input for CloVR-Comparative. Colored boxes and phylogroup assignments were manually added to the automatically generated tree in Newick format

The increased resolution of whole-genome alignment-based phylogenetic analyses compared to multi-locus sequence typing (MLST) or related methods that rely on a relatively small set of genomic marker loci has been demonstrated previously [17–19]. In order to evaluate the phylogenetic predictions from CloVR-Comparative, it was tested on a set of 40 *E. coli* genomes from different phylogroups (A, B1, B2, B2A, D, E, SF, and SS), which was also used in a recent publication that compared whole-genome sequence typing with multi-locus sequence typing (MLST) [9]. CloVR-Comparative identified a core genome length of 2742 kbp and predicted a phylogenetic tree that was consistent with the recent publication and correctly clustered and separated the different *E. coli* phylogroups (Fig. 4).

**Fig. 5:**
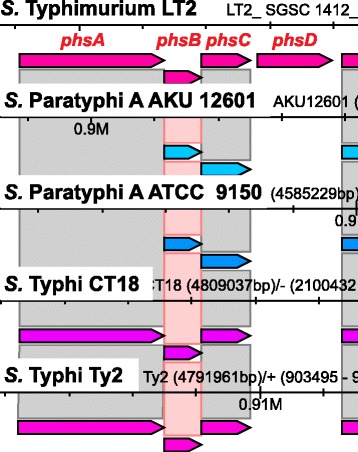
Screenshot of the *phsABCD* gene cluster comparison between different *S. enterica* serotypes. The screenshot from the Sybil comparative analysis tool highlights the *phs* operon that encodes the enzymes for the anaerobic production of hydrogen sulfide from thiosulfate, which are used in anaerobic respiration. The comparison shows that of the four genes that are present in *S.* Typhimurium LT2, two (*phsA* and *phsD*) are missing from the two *S.* Paratyphi A strains AKU 12601 and ATCC 9150 and one (*phsD*) from the two *S.* Typhi strains CT18 and Ty2. The corresponding genomic regions were manually checked and confirmed to contain interrupted open reading frames in those genomes without gene calls. Gene designations in red were manually added to the screenshot that was directly copied from Sybil browser

**Fig. 6:**
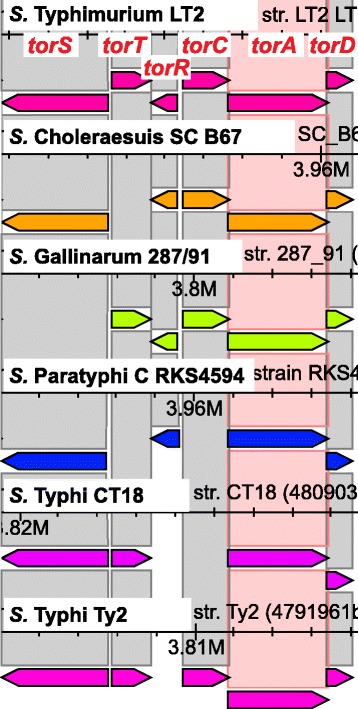
Screenshot of the *torSTRCAD* gene cluster comparison between different *S. enterica* serotypes. The Sybil screenshot highlights the *tor* gene cluster that is responsible for the reduction of trimethylamine oxide (TMAO) to trimethylamine, which is used in anaerobic respiration. The comparison shows that of the six genes that are present in *S.* Typhimurium LT2 at least one, in several cases two are missing from *S.* Choleraesuis SC B67 (*torT*), *S.* Gallinarum 287/91 (*torS*), *S.* Paratyphi A RKS4594 (*torTR*), *S.* Typhi CT18 (*torRC*) and Ty2 (*torR*). The corresponding genomic regions were manually checked and confirmed to contain interrupted open reading frames in those genomes without gene calls. Gene designations in *red* were manually added to the screenshot that was directly copied from Sybil browser. Gene designations in *red* were manually added to the screenshot that was directly copied from Sybil browser

The species *S. enterica* for which more than 2,500 serotypes have been described, is characterized by an unusual phenotypic diversity with respect to virulence, host adaptation and antibiotic resistance. We could previously show, using a comparative analysis of translated CDSs by BLAST Score Ratio [20], that distinct sublineages within the *S. enterica* species, represented by serotypes, appear to have lost specific metabolic capabilities compared to their last common ancestor [21]. Here we ran CloVR-Comparative on the same set of 28 characterized *S. enterica* genomes. Using Sybil to search through the CloVR-Comparative output, we confirmed previously identified *S. enterica* genotypes, such as the absence of genes encoding key elements of anaerobic respiration pathways responsible for the use of alternative terminal electron acceptors other than O2 under anoxic conditions [22]. For example, three different CDSs required for thiosulfate reduction are absent from the typhoid *S. enterica* serotype Typhi strains CT18 and Ty2 (*phsD*) and serotype Paratyphi A strains AKU 12601 and ATCC 9150 (*phsAD*) (Fig. 5). Similarly, different CDSs of the gene cluster that is responsible for the ability to reduce trimethylamine N-oxide (TMAO) are lost in the genomes of *S.* Choleraesuis strain SC B67 (*torT*), *S.* Gallinarum strain 287/91 (*torS*), *S.* Paratyphi C strain RKS4594 (*torCT*) and *S.* Typhi strains CT18 (*torCR*) and Ty2 (*torR*) (Fig. 6).

### Comparison to other microbial comparative genomics tools and services

Several other bioinformatics platforms for comparative microbial genomics have become available in recent years. Built on top of server-hosted databases of microbial genomes or pre-computed sets of orthologous genes, services like EDGAR [23], IMG [24], INSYGHT [25], MGBD [26], and MicrobesOnline [27] mostly provide support in the form of web-based workbenches, similar to the CloVR Dashboard. INSYGHT or other programs such as CMG-biotools [28] are also available for download and local installation, using a similar virtual machine-based framework as CloVR. Users of online services benefit from free computational resources, albeit with limited control over uploaded data or wait times for analyses to be started and completed. The CloVR framework [1] provides full flexibility over the computational infrastructure used, while maximizing control over user data. With sufficient local hardware support CloVR-Comparative can run for free and without transfer of user data over the Internet. The Amazon EC2 cloud, while charging for leased computational resources, enables users with only a computer with an Internet connection to run analyses that they otherwise would not have access to.

A direct comparison of available tools is difficult, as they rely on different analysis methods (e.g. based on comparisons of whole-genomes, genes, codon usages or nucleotide sequence compositions) and put different weight on functionality, configurability and visualization. It should be noted that whole-genome alignments as performed by CloVR-Comparative provide a very high resolution for phylogenetic analyses of closely related organisms. Comparisons of gene and protein sequences as performed for example by INSYGHT or of codon or amino acid usages, as performed by CMG-biotools on the other hand are well-suited to analyze more divergent genomes. By limiting the number of configurable options, fully automating the analysis protocol and providing seamless cloud support, as afforded by the CloVR framework, CloVR-Comparative puts a strong emphasis on user-friendliness and access of inexperienced users to comparative genomics.

Another advantage of CloVR-Comparative is its close integration with other CloVR-supported automated analysis protocols. Within the CloVR virtual appliance computational support is provided for the entire bioinformatics process of genome projects, from raw sequence assembly, gene prediction and functional annotation with CloVR-Microbe [3] to comparative analyses of private or public genomes with CloVR-Comparative.

### Scalability, runtime and cost

As the whole-genome alignment step of the CloVR-Comparative analysis protocol is memory-intensive, leasable cloud services such as Amazon’s EC2 with flexibility over the instance type and corresponding CPU and memory setups can represent a valuable alternative to local runs of CloVR-Comparative. To evaluate the performance of CloVR-Comparative and provide benchmarks on computational requirements, runtimes and costs, several test datasets (Table 1) were run and compared on local desktop computers and different EC2 instance types (Table 2). Runtimes were determined based on processing times recorded with the Ergatis workflow system [29] that is part of the CloVR framework and used for process management. With the exception of the analysis of 40 *E. coli* genomes, all runs finished in around 24 h (*E. coli* run: 57 h), at a cost of ~ $14 or less (*E. coli* cost: $19.89). All runs could be completed on relatively modest-size local desktop computers with 2–8 CPUs and 8–16 GB of RAM. Memory requirements and runtimes are largely determined by the whole-genome alignment and therefore dependent on both the input genome lengths and the sequence similarity and synteny of the input genomes. As a consequence running CloVR-Comparative on a large number of draft genomes with many contigs is going to increase runtime and memory requirements.

**Table 1:** Input test datasets for CloVR-Comparative

Species	Genomes	Size +/− SD [Mbp]	Core genome [Kbp]	COGs
*Neisseria meningitidis*	5	2.19	1775.09	1534
*Legionella pneumophila*	5	3.46	2838.89	2443
*Yersinia pestis*	12	4.58	4076.66	2793
*Helicobacter pylori*	25	1.63	1294.81	1081
*Streptococcus pneumoniae*	34	2.13	1312.15	742
*Salmonella enterica*	28	4.78	3371.09	2893
*Escherichia coli*	40	5.05	2744.19	1708

**Table 2:** Resources, runtimes and costs

	Amazon EC2			Local desktop	
Dataset	Instance type	Runtime [hours]	Cost	Available resources	Runtime [hours]
*Neisseria meningitidis*	c1.xlarge	3.45	$1.79	2 CPUs, 8GB RAM	3.15
*Legionella pneumophila*	c1.xlarge	3.65	$1.90	2 CPU, 8GB RAM	3.67
*Yersinia pestis*	c1.xlarge	6.97	$3.62	2 CPUs, 8GB RAM	6.70
*Helicobacter pylori*	c1.xlarge	17.08	$8.88	2 CPUs, 8GB RAM	17.72
*Streptococcus pneumoniae*	c1.xlarge	16.87	$8.77	2 CPUs, 8GB RAM	15.98
*Salmonella enterica*	c1.xlarge	26.10	$13.57	2 CPUs, 8GB RAM	24.42
*Escherichia coli*	m1.xlarge	56.83	$19.89	4 CPUs, 16GB RAM^a^	35.07

Amazon EC instance types

c1.xlarge, previous generation: 8 virtual CPUs, 7GB RAM, at $0.520 per hour

m1.xlarge, previous generation: 4 virtual CPUs, 15GB RAM, at, $0.350 per hour

^a^The local setting for the *E. coli* run was simulated on a server running VMware ESX, using a virtual instance with the listed CPU and memory allocations

It is important to note that even with cloud support and large instance types, CloVR-Comparative is not going to be able to process the hundreds or thousands of genomes that are now often generated in large-scale genome projects. However, it is also unlikely that other bioinformatics tools or algorithms will be able to keep up with the continuous increase in available genome sequence data for comparable analyses. To address this bioinformatics bottleneck, a multi-step process seems more suitable whereby genomes of interest are first compared against a modest-size subset of 20–50 reference genomes and in a second step this preliminary analysis is used to inform fine-tuned subsequent analyses of genome subsets.

## Conclusions

CloVR-Comparative is an open-source automated, microbial sequence analysis tool for reference-free comparative genome analysis that allows researchers without bioinformatics experience and local computational support to easily perform analyses of up to 50 genomes. To our knowledge this is the first bioinformatics tool in the field that combines broad functionality with the user-friendliness of a graphical user interface and the independence of local computational resources, and therefore has the potential to increase the value of microbial genome sequence data to the community.

## Availability and requirements

CloVR-Comparative is freely available as part of the CloVR software (http://www.clovr.org). CloVR runs on Windows 7 or higher, Mac OSX and Linux (Ubuntu, RedHat, CentOS, Fedora) or any distribution that can be used to run VMware (http://www.vmware.com/) or VirtualBox (https://www.virtualbox.org/) virtualization software. All genome sequence datasets that were analyzed as part of this study are publicly available and accession numbers to retrieve the sequence data have been included or referenced in the text. Minimum requirements to run CloVR-Comparative are the following: a CPU with at least 4 cores, 8GB of memory and 100GB hard drive space.

- **Project name:** CloVR
- **Project home page:** http://www.clovr.org
- **Operating system(s):** Platform Independent
- **Programming language:** Python
- **Other requirements:** VMware or VirtualBox virtual machine players
- **License:** BSD
- **Any restrictions to use by non-academics:** No

## Additional files

Additional file 1: Table S1. List of components and scripts used by CloVR-Comparative, including links to corresponding software repositories. (TXT 2 kb)

Additional file 2: Table S2. Example of a summary report from the comparative analysis of 40 *E. coli* genomes as listed in Table 1. (TXT 3 kb)

## Abbreviations

CDS: Coding sequences
CloVR: Cloud virtual resource
COG: Cluster of orthologous genes
GUI: Graphical user interface
IT: Information technology
NCBI: National center for biotechnology information
SNP: Single nucleotide polymorphism

## References

[1] Angiuoli SV, Matalka M, Gussman A, Galens K, Vangala M, Riley DR, Arze C, White JR, White O, Fricke WF (2011). CloVR: A virtual machine for automated and portable sequence analysis from the desktop using cloud computing. BMC Bioinformatics.

[2] Angiuoli SV, White JR, Matalka M, White O, Fricke WF (2011). Resources and costs for microbial sequence analysis evaluated using virtual machines and cloud computing. PLoS One.

[3] Galens K, White JR, Arze C, Matalka M, Giglio MG, Team TC, Angiuoli SV, Fricke WF (2011). CloVR-Microbe: Assembly, gene finding and functional annotation of raw sequence data from single microbial genome projects – standard operating procedure, version 1.0. Nature Preceding.

[4] Angiuoli SV, Salzberg SL (2011). Mugsy: fast multiple alignment of closely related whole genomes. Bioinformatics.

[5] Kurtz S, Phillippy A, Delcher AL, Smoot M, Shumway M, Antonescu C, Salzberg SL (2004). Versatile and open software for comparing large genomes. Genome Biol.

[6] Angiuoli SV, Dunning Hotopp JC, Salzberg SL, Tettelin H (2011). Improving pan-genome annotation using whole genome multiple alignment. BMC Bioinformatics.

[7] Edgar RC (2004). MUSCLE: multiple sequence alignment with high accuracy and high throughput. Nucleic Acids Res.

[8] Riley DR, Angiuoli SV, Crabtree J, Dunning Hotopp JC, Tettelin H (2012). Using Sybil for interactive comparative genomics of microbes on the web. Bioinformatics.

[9] Sahl JW, Matalka MN, Rasko DA (2012). Phylomark, a tool to identify conserved phylogenetic markers from whole-genome alignments. Appl Environ Microbiol.

[10] Price MN, Dehal PS, Arkin AP (2010). FastTree 2--approximately maximum-likelihood trees for large alignments. PLoS One.

[11] Crabtree J, Agrawal S, Mahurkar A, Myers GS, Rasko DA, White O (2014). Circleator: flexible circular visualization of genome-associated data with BioPerl and SVG. Bioinformatics.

[12] Croucher NJ, Didelot X (2015). The application of genomics to tracing bacterial pathogen transmission. Curr Opin Microbiol.

[13] Brzuszkiewicz E, Thurmer A, Schuldes J, Leimbach A, Liesegang H, Meyer FD, Boelter J, Petersen H, Gottschalk G, Daniel R (2011). Genome sequence analyses of two isolates from the recent Escherichia coli outbreak in Germany reveal the emergence of a new pathotype: Entero-Aggregative-Haemorrhagic Escherichia coli (EAHEC). Arch Microbiol.

[14] Rohde H, Qin J, Cui Y, Li D, Loman NJ, Hentschke M, Chen W, Pu F, Peng Y, Li J (2011). Open-source genomic analysis of Shiga-toxin-producing E. coli O104:H4. N Engl J Med.

[15] Rasko DA, Webster DR, Sahl JW, Bashir A, Boisen N, Scheutz F, Paxinos EE, Sebra R, Chin CS, Iliopoulos D (2011). Origins of the E. coli strain causing an outbreak of hemolytic-uremic syndrome in Germany. N Engl J Med.

[16] Kingsley RA, Kay S, Connor T, Barquist L, Sait L, Holt KE, Sivaraman K, Wileman T, Goulding D, Clare S (2013). Genome and transcriptome adaptation accompanying emergence of the definitive type 2 host-restricted Salmonella enterica serovar Typhimurium pathovar. MBio.

[17] Sahl JW, Steinsland H, Redman JC, Angiuoli SV, Nataro JP, Sommerfelt H, Rasko DA (2011). A comparative genomic analysis of diverse clonal types of enterotoxigenic Escherichia coli reveals pathovar-specific conservation. Infect Immun.

[18] Gardy JL, Johnston JC, Ho Sui SJ, Cook VJ, Shah L, Brodkin E, Rempel S, Moore R, Zhao Y, Holt R (2011). Whole-genome sequencing and social-network analysis of a tuberculosis outbreak. N Engl J Med.

[19] Harris SR, Cartwright EJ, Torok ME, Holden MT, Brown NM, Ogilvy-Stuart AL, Ellington MJ, Quail MA, Bentley SD, Parkhill J (2013). Whole-genome sequencing for analysis of an outbreak of meticillin-resistant Staphylococcus aureus: a descriptive study. Lancet Infect Dis.

[20] Rasko DA, Myers GS, Ravel J (2005). Visualization of comparative genomic analyses by BLAST score ratio. BMC Bioinformatics.

[21] Fricke WF, Mammel MK, McDermott PF, Tartera C, White DG, Leclerc JE, Ravel J, Cebula TA (2011). Comparative genomics of 28 Salmonella enterica isolates: evidence for CRISPR-mediated adaptive sublineage evolution. J Bacteriol.

[22] Winter SE, Baumler AJ (2011). A breathtaking feat: to compete with the gut microbiota, Salmonella drives its host to provide a respiratory electron acceptor. Gut Microbes.

[23] Blom J, Kreis J, Spanig S, Juhre T, Bertelli C, Ernst C, Goesmann A (2016). EDGAR 2.0: an enhanced software platform for comparative gene content analyses. Nucleic Acids Res.

[24] Markowitz VM, Chen IM, Palaniappan K, Chu K, Szeto E, Grechkin Y, Ratner A, Jacob B, Huang J, Williams P (2012). IMG: the Integrated Microbial Genomes database and comparative analysis system. Nucleic Acids Res.

[25] Lacroix T, Therond S, Rugeri M, Nicolas P, Gendrault A, Loux V, Gibrat JF (2016). Synchronized navigation and comparative analyses across Ensembl complete bacterial genomes with INSYGHT. Bioinformatics.

[26] Uchiyama I, Mihara M, Nishide H, Chiba H (2015). MBGD update 2015: microbial genome database for flexible ortholog analysis utilizing a diverse set of genomic data. Nucleic Acids Res.

[27] Dehal PS, Joachimiak MP, Price MN, Bates JT, Baumohl JK, Chivian D, Friedland GD, Huang KH, Keller K, Novichkov PS (2010). MicrobesOnline: an integrated portal for comparative and functional genomics. Nucleic Acids Res.

[28] Vesth T, Lagesen K, Acar O, Ussery D (2013). CMG-biotools, a free workbench for basic comparative microbial genomics. PLoS One.

[29] Orvis J, Crabtree J, Galens K, Gussman A, Inman JM, Lee E, Nampally S, Riley D, Sundaram JP, Felix V (2010). Ergatis: a web interface and scalable software system for bioinformatics workflows. Bioinformatics.

